# Ginsenoside Rb1 Attenuates Intestinal Ischemia Reperfusion Induced Renal Injury by Activating Nrf2/ARE Pathway

**DOI:** 10.3390/molecules17067195

**Published:** 2012-06-12

**Authors:** Qian Sun, Qing-Tao Meng, Ying Jiang, Zhong-Yuan Xia

**Affiliations:** Department of Anesthesiology, Renmin Hospital of Wuhan University, 238 Jiefang Road, Wuhan, Hubei 430060, China

**Keywords:** ginsenoside Rb1, NF-E2-related factor-2, renal injury, intestinal ischemia reperfusion, ho-1

## Abstract

Intestinal ischemia reperfusion (IIR) is a serious clinical condition associated with simultaneous multiple organ dysfunction. The aim of this study was to investigate the effects of ginsenoside Rb1 on IIR induced renal injury in mice. An intestinal ischemia reperfusion mouse model was established by superior mesenteric artery (SMA) occlusion for 45 min, followed by reperfusion for 2 h. IIR induced renal injury characterized by increase of BUN, Cr and NGAL in serum, MDA levels and decrease of SOD levels in the renal tissues. Ginsenoside Rb1 (30, 60 mg/kg) given intraperitoneally before reperfusion attennuated renal injury, which was associated with decrease of BUN, Cr and NGAL in serum, MDA levels and increase of SOD levels in the renal tissues. Furthermore, the immunohistochemistry and Western blot data showed that ginsenoside Rb1 dramatically reversed IIR induced renal injury, associated with upregulated nuclear factor erythroid 2-related factor 2 (Nrf2) and heme oxygenase-1 (HO-1) in renal tissues. Our data suggests that ginsenoside Rb1 attenuates acute renal injury induced by intestinal ischemia reperfusion by activating the Nrf2/ARE pathway.

## 1. Introduction

Intestinal ischemia reperfusion (IIR) is a serious clinical condition associated with simultaneous multiple organ dysfunction syndrome (MODS) [1]. Multiple organ failure is a frequent complication after intestinal ischemia reperfusion and involves organs like the lung [2], liver [3], heart [4] and kidney [5]. The kidney is an important organ affected by intestinal ischemia reperfusion because of the hypotension and oxidative stress resulting from the reperfused intestine [6]. However, the mechanisms responsible for the renal injury caused by IIR are not clearly defined.

The pathogenesis of MODS after IIR is multiple, but the key point is the development of oxidative stress with an inflammatory response [7]. A generalized microvascular injury induced by various cytotoxic agents (oxygen-derived free radicals [8], neutrophils and cytokines [7]) has been previously implicated. However, recent studies have shown the HO-1 mediators are regulated by transcription factor Nrf2. Upon exposure of cells to oxidative stress and electrophilic chemical insults, Nrf2 activity is markedly increased. The signal-dependent activation of Nrf2 function is a complex phenomenon, and the precise mechanisms involved remain a subject of intensive investigation [9,10,11]. So we hypothesised that Nrf2 signal activation was the key mechanism responsible for the renal injury caused by IIR. Ginseng is believed to be the most unique traditional medical herbs because it contains the maximum numbers of active constituents, has the most extensive pharmacological effects and specific mechanism of actions in materia medica. Ginsenoside Rb1, one of the panaxadiols, showed anti-stress effects in acute, chronic, and repeated stress models [12]. It has beneficial effects on lung [13], cerebral [14], liver [15] and cardiomyocyte [16] ischemia reperfusion injury, but the effect of ginsenoside Rb1 on IIR injury and renal injury induced by IIR has not been reported. In this paper, we used an intestinal IR model to investigate the influence of ginsenoside Rb1 on renal injury and Nrf2/ARE activity induced by IIR.

## 2. Results and Discussion

### 2.1. Effects of Ginsenoside Rb1 on Renal Functional Injury Induced by IIR

BUN and Cr are two important indexes and play an important role in renal functional injury after IIR. NGAL is arguably the most promising emerging biomarker for detection of AKI. Studies in mouse models of renal ischemia reperfusion showed NGAL was highly up-regulated at an early stage [17]. Importantly, NGAL protein is easily detected in the blood and urine soon after AKI in pre-clinical studies [18,19]. As seen in Table 1, following 45 min of intestinal ischemia, reperfusion significantly increased serum BUN, Cr and NAGL levels in the IIR group compared with the sham group (*p* < 0.01).

**Table 1:** Changes of serum BUN (mmol/L), Cr (μmol/L) and NGAL (ng/ml) levels.

	Sham	IIR	NS	Rb1-30	Rb1-60
BUN	9.37 ± 1.19	21.55 ± 1.50 *	18.87 ± 2.16 *	15.52 ± 1.23 ^#^	12.91 ± 1.41 ^#^
Cr	10.26 ± 1.25	20.65 ± 1.53 *	19.53 ± 1.19 *	16.00 ± 1.50 ^#^	13.14 ± 1.22 ^#^
NAGL	71.65 ± 4.65	155.93 ± 8.07 *	150.11 ± 6.09 *	124.44 ± 6.52 ^#^	101.76 ± 12.20 ^#^

Data are means ± SD from ten mice per group. * *p* < 0.05 *vs.* sham group, ^#^ *p* < 0.05 *vs.* IIR group. Treatment with 30 mg/kg and 60 mg/kg ginsenoside Rb1 could markedly decrease serum BUN, Cr and NAGL levels compared with both the IIR group (*p* < 0.05) and NS group (*p* < 0.05).

### 2.2. Effects of Ginsenoside Rb1 on SOD and MDA Levels in Renal Tissues

**Figure 1:**
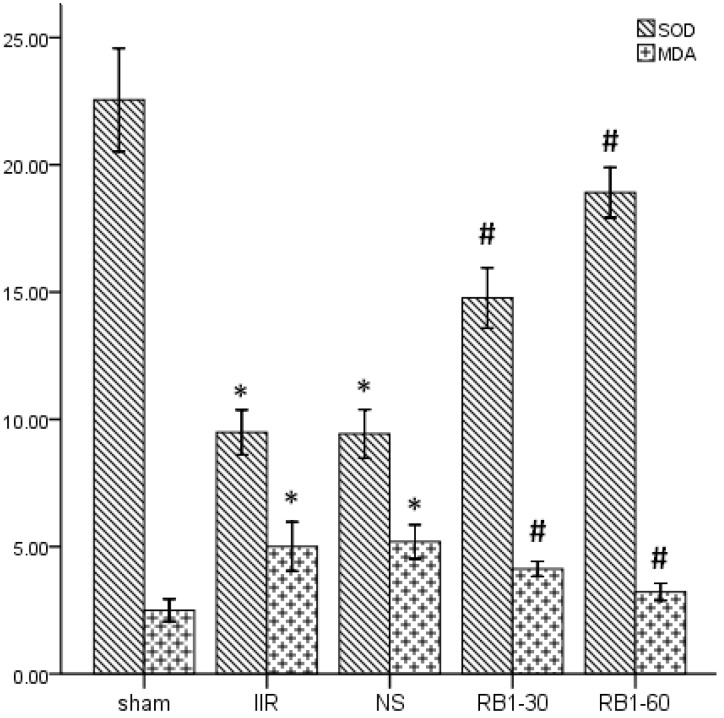
Changes of levels of SOD and MDA in renal tissues. * *p* < 0.05 *vs.* sham group, ^#^ *p* < 0.05 *vs.* IIR group.

Oxidative stress mediators such as reactive oxygen species (ROS), which cause lipid peroxidation and protein oxidation, are suggested to play a crucial role in ischemia reperfusion damage [20,21]. SOD is an oxidoreductase that catalyzes the reaction between superoxide anions and hydrogen to yield molecular oxygen and hydrogen peroxide. Large numbers of oxygen free radicals are generated during periods of ischemia followed by reperfusion, leading to excessive SOD consumption [22]. MDA is a naturally occurring product of lipid peroxidation and prostaglandin biosynthesis that is mutagenic and carcinogenic [23]. MDA can be dosed in both tissue and blood and its concentration is directly proportional to the cell damage caused by free radicals [24]. As shown in Figure 1, following 45 min of intestinal ischemia, reperfusion significantly decreased renal tissues SOD (U/mg pro) and increased MDA (nmol/g pro) levels in the IIR group compared with those in sham group (*p* < 0.05). Administration of 30 mg/kg and 60 mg/kg ginsenoside Rb1 increased renal tissues SOD and decreased MDA levels compared with the IIR group (*p* < 0.05). Results indicated that IIR induced free radical damage in renal tissues and ginsenoside Rb1 could lighten renal injury due to their antioxidative properties.

### 2.3. Effects of Ginsenoside Rb1 on HO-1 and Nrf2 Expressions in Renal Tissues by Immunohisto- Chemical Analysis

**Figure 2:**
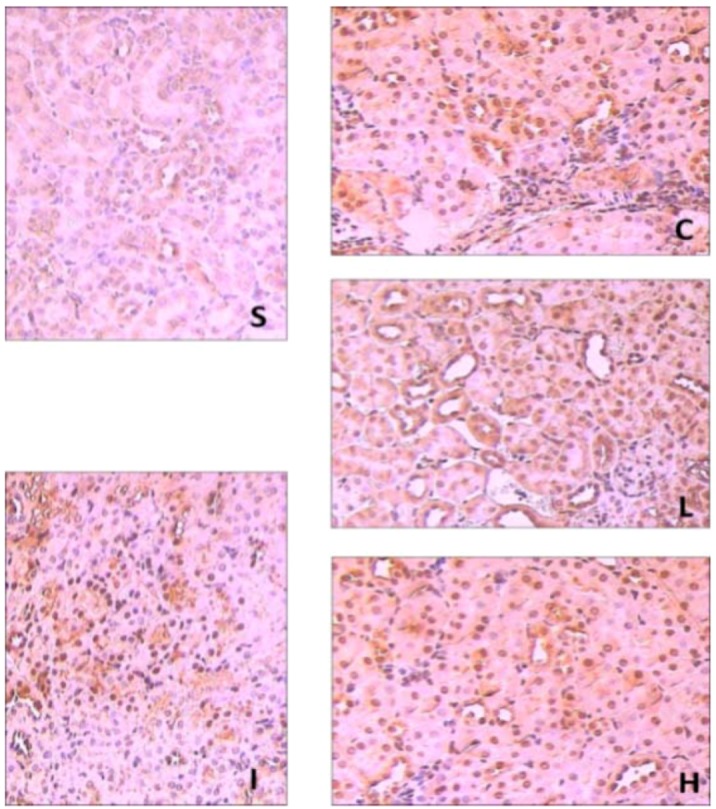
The expression of Nrf2 in the renal tissues in the groups by immunohistochemmistry (SP × 200). S: Sham group; I: IIR group; C: NS group; L: RB1-30 group; H: RB1-60 group.

**Figure 3:**
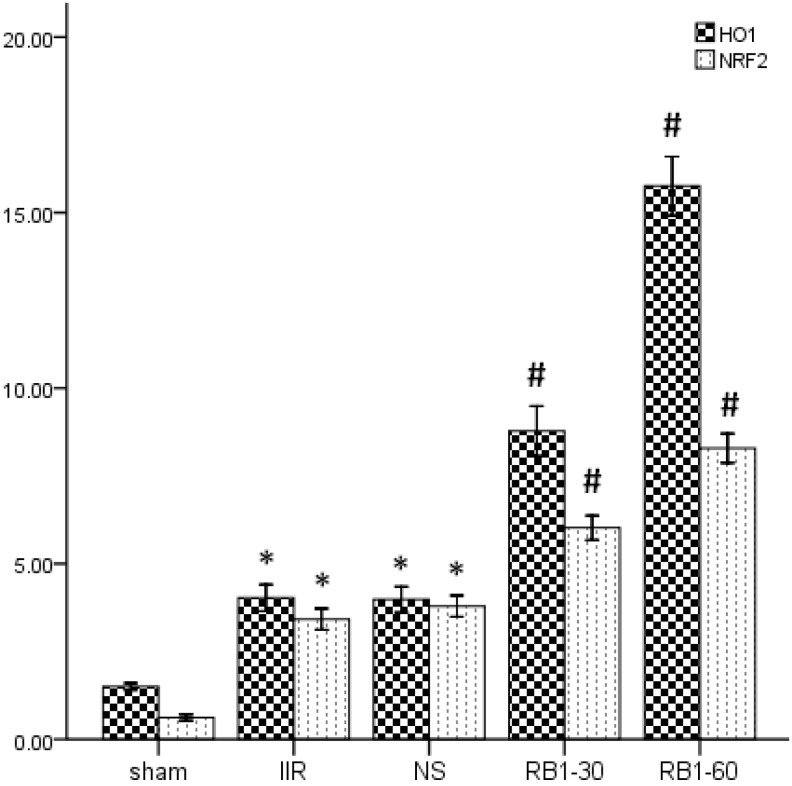
Immunohistochemical staining of HO-1 and Nrf2 expressions in the renal tissues in the groups (IOD × 10^3^). * *p* < 0.05 *vs.* sham group, ^#^*p* < 0.05 *vs.* IIR group.

Recent research has shown that Nrf2 has multiple functions including acute, transient stress responses to oxidative insults [25]. There are many evidences supporting the protective role of the Nrf2-mediated pathway against oxidative stress and inflammation. However, Nrf2 could be activated by oxidative stress, and then translocated into the nucleus to perform tasks. The intermolecular correlation between activated Nrf2 and ARE induce the expressions of antioxidant proteins and phase II enzymes which act actively in cellular defense systems [26,27]. Thus, Nrf2 is important for protecting cells and multiple tissues by coordinately up-regulating ARE-related detoxification and antioxidant genes and molecules required for the defense system in each specific environment [28]. As shown in Figure 2 and Figure 3, the expression of Nrf2 in the sham group showed light brown immunostaining in cytoplasm and no staining in the nuclei. A significantly positive expression of Nrf2 evidenced by a strong brown staining in cytoplasm and nuclei were observed in the IIR group (*p* < 0.05) and NS group (*p* < 0.05). Compared with the IIR group, the positive expression of Nrf2 increased significantly in RB1-30 group (*p* < 0.05) and RB1-60 group (*p* < 0.05).

**Figure 4:**
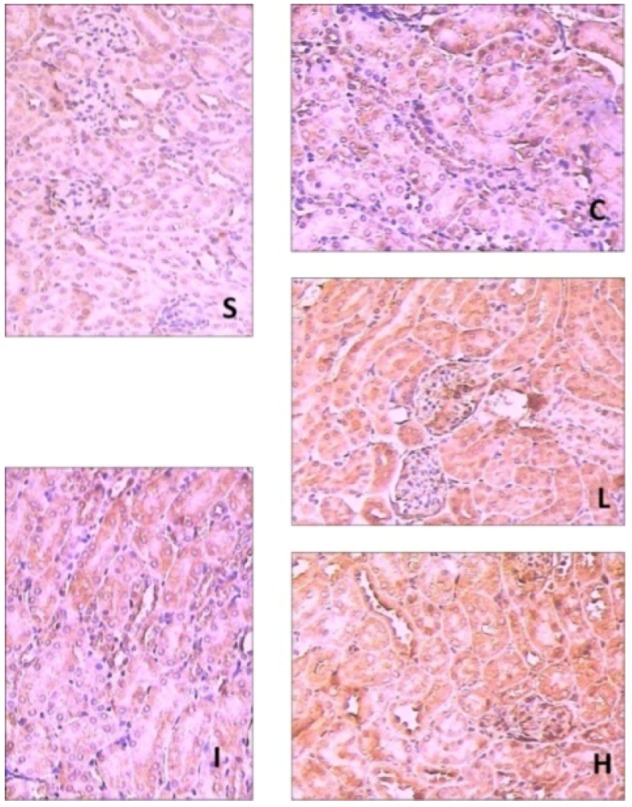
The expression of HO-1 in the renal tissues in the groups by immunohistochemmistry (SP × 200). S: Sham group; I: IIR group; C: NS group; L: RB1-30 group; H: RB1-60 group.

And shown in Figure 3 and Figure 4, the expression of HO-1 in sham group showed little brown immunostaining in cytoplasm, while significant positive expression of HO-1 observed as brown staining in cytoplasm was observed in the IIR group (*p* < 0.05) and NS group (*p* < 0.05). Compared with the IIR group, the positive expression of HO-1 increased significantly in cytoplasm in RB1-30 group (*p* < 0.05) and RB1-60 group (*p* < 0.05).

Our results indicated that HO-1 could be upregulated by ginsenoside Rb1 treatment through Nrf2/ARE pathway. Although HO-1 does not directly lead to an antioxidant reaction, its induction may consider to be a cytoprotective response against the oxidative stress [29].

### 2.4. Effects of Ginsenoside Rb1 on HO-1 and Nrf2 Expressions in Renal Tissues by Western Blot Analysis

**Figure 5:**
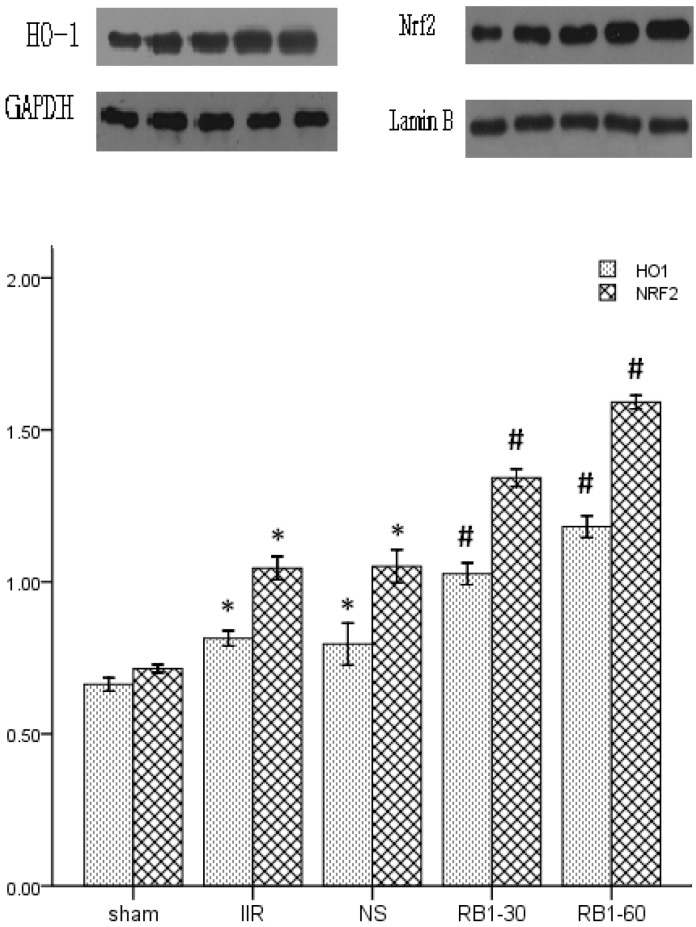
The expressions of Nrf2 and HO-1 in the renal tissues in the groups by western blot. * *p* < 0.05 *vs.* sham group, ^#^ *p* < 0.05 *vs.* IIR group.

In our research, we paid attention to the effect of ginsenoside Rb1 on activating the Nrf2/ARE pathway in renal injury induced by IIR. We found that ginsenoside Rb1 attenuated renal injury induced by IIR, which was characterized by improved renal tissues pathology and renal function. The Nrf2 expression increased, paralleling the enhanced antioxidant capacity, but not the inflammatory reaction in renal tissues. HO-1 is highly expressed by oxidative stress, ischemia-reperfusion, cytokines, nitric oxide, bacterial lipopolysaccharide (LPS) [30]. The translocation of Nrf2 is considered a major defense mechanism that plays a key role in the induction of HO-1 [31]. As shown in Figure 5, Western blot analysis showed weak Nrf2/HO-1 positive signals in the renal tissues in sham group. In contrast, significant increase of the Nrf2 and HO-1 protein expressions were found in IIR group (*p* < 0.05). Compared with the IIR group, the signals were strengthened in the Rb1-30 group (*p* < 0.05) and Rb1-60 group (*p* < 0.05).

### 2.5. Renal Histopathological Assessment

**Figure 6:**
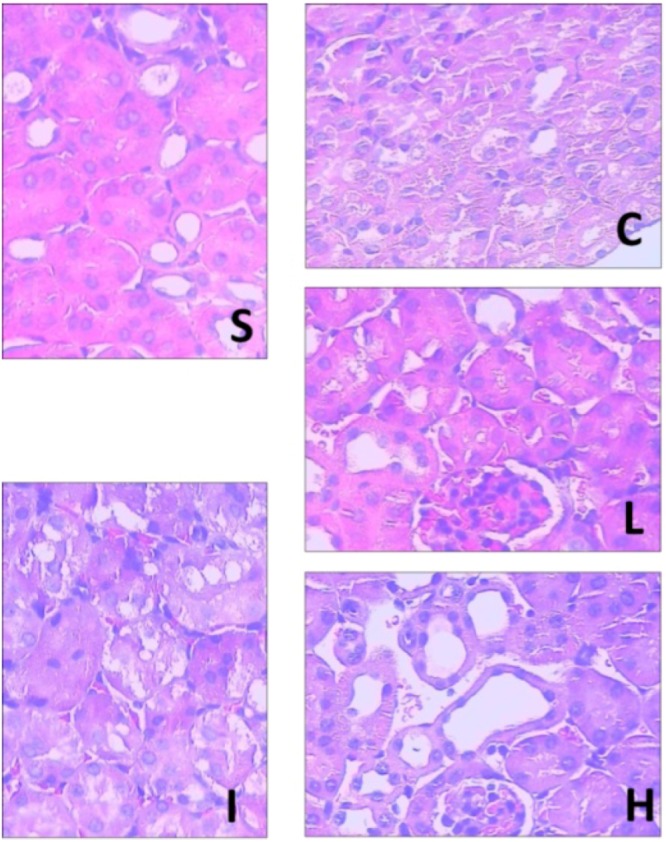
Pathological pictures of the renal tissues in the groups (HE × 400). S: Sham group; I: IIR group; C: NS group; L: RB1-30 group; H: RB1-60 group.

Ginsenoside Rb1 could alleviate renal histology injury at 2 h after intestinal reperfusion. In Figure 6, the renal tubules in the IIR group show pathological changes, including oedema, necrosis and vacuolization. There is little amelioration in the NS group. A significant amelioration of histological oedema, necrosis and vacuolization was seen in the 30 mg/kg and 60 mg/kg ginsenoside Rb1 treated groups. 

When compared with the total severity scores measured from kidneys obtained from sham animals (16.70 ± 4.06), IIR group (198.60 ± 9.71) and NS group (196.80 ± 9.20) produced significant increase in total severity score (*p* < 0.05), which was significantly reduced by administration of RB1-30 group (135.10 ± 8.71) and RB1-60 group (94.60 ± 9.24) (*p* < 0.05).

## 3. Experimental

### 3.1. Materials

Adult male C57BL/6J mice, weighing 25 ± 3 g, were obtained from HUNAN SLAC JD Laboratory Animal Co. Ltd. This study complied with the European Community Guidelines for the CARE and Use of Experimental Animals and was approved by the Animal Research Committee of Wuhan University (Wuhan, China). Ginsenoside Rb1 (99.5%) was obtained from the Research Center of Traditional Chinese Medicine (Wuhan, China) and dissolved in saline. The Superoxide Dismutase (SOD) and Malondialdehyde (MDA) assay kits were obtained from Nanjing Jiancheng Bioengineering Institute (Nanjing, China). Neutrophil gelatinase-associated lipocalin (NGAL) ELISA assay kits were obtained from Boster Biological Technology (Wuhan, China). Antibodies for Nrf2 and HO-1 were purchased from Santa Cruz Biotechnology, Inc (Santa Cruz, CA, USA). All other chemicals were of the highest grade available commercially.

### 3.2. Experimental Protocol

The animals were anesthetized with sodium pentobarbital (50 mg/kg) intraperitoneally. The IIR model was established by SMA occlusion [32]. Mice were assigned randomly into one of five experimental groups (n = 10 in each group) as follows: (i) a sham- operated group (sham group) that underwent isolation of the SMA without occlusion; (ii) an intestinal ischemia reperfusion group (IIR group) subjected to 45 min intestinal ischemia and 2 h reperfusion after the SMA had been isolated and occluded; (iii) a group treated with 10 mL/kg saline 10 min before reperfusion (NS group); (iv) a 30 mg/kg ginsenoside Rb1 (3 mg/mL, dissolved in saline, 10 mL/kg) treated group (Rb1-30 group), in which surgery was performed as in the IIR group with administration of 10 ml/kg 3% ginsenoside Rb1 intraperitoneally 10 min before reperfusion; and (v) a 60 mg/kg ginsenoside Rb1 (6 mg/mL, dissolved in saline, 10 mL/kg) treated group (Rb1-60 group), in which surgery was performed as in the IIR group with administration of 10 mL/kg 6% ginsenoside Rb1 intraperitoneally 10 min before reperfusion. Kidneys and blood samples were obtained for analysis at the end of the 2 h reperfusion period.

### 3.3. Measurement of Serum BUN, Cr and NGAL

Serum levels of Creatinine (Cr), Urea Nitrogen (UN) were measured using an Olympus automatic analyzer (AU5400; Olympus Optical, Tokyo, Japan). NGAL level was measured using ELISA assay kit according to the manufacturer’s instructions.

### 3.4. Renal Tissues SOD and MDA Assay

The renal tissues were harvested and immediately homogenized on ice in 5 volumes of normal saline. The homogenates were centrifuged at 1,200 g for 10 min. The SOD and MDA levels in the supernatant were measured using SOD and MDA assay kits according to the manufacturer’s instructions.

### 3.5. Renal HO-1 and Nrf2 Immunohistochemical Assays

Paraffin-embedded renal sections were stained using the Strept Avidin-Biotin Complex (SP) immunohistochemistry technique for HO-1 and Nrf2 detection. Brown staining in the cytoplasm and/or nucleus was considered an indicator of positive expression. With the Image-Pro® Plus version 6.0, results were evaluated semiquantitatively according to optical density values of positive expression.

### 3.6. Renal Tissues HO-1 and Nrf2 Western Blot Analysis

Endochylema and cellular nuclear proteins were extracted from frozen renal tissue with a nuclear extract kit according to the manufacturer’s instructions. An equal amount of protein was loaded onto 12% SDS-PAGE at 100 V for 3 h. After electrophoresis, proteins were transferred onto PVDF membranes at 200 mA for 2 h. The transferred membranes were incubated overnight at 4 °C with rabbit polyclonal antibodies HO-1 or Nrf2 (1:800 dilution) against mouse in TBS-T containing 5% skim milk. After washing three times in Tris phosphate-buffered sodium (TBS-T), membranes were incubated with antirabbit IgG conjugated to HRP at a dilution of 1:2,000 in TBS-T containing 5% skim milk for 2 h at room temperature. The immunoreactive bands were visualized with enhanced chemiluminescence (ECL) and captured on X-ray film. Optical density of the bands was measured with BandScan imaging analysis system.

### 3.7. Renal Histopathological Assessment

The left kidney sections were stained with haematoxylin and eosin for the observation of renal tissues structure (BX50; Olympus Optical, Tokyo, Japan). Histologic assessment of tubular necrosis was determined semiquantitatively using a method modified from McWhinnie *et al.* [33]. A score from 0 to 3 was given for each tubular profile involving an intersection: 0 normal histology; 1 tubular cell swelling, brush border loss, nuclear condensation, with up to one third of the tubular profile showing nuclear loss; 2 same as for score 1, but greater than one third and less than two thirds of the tubular profile show nuclear loss; and 3, greater than two thirds of the tubular profile showing nuclear loss.

### 3.8. Statistical Analysis

Mean ± S.D. values were calculated to summarize all outcome measurements. One-way analysis of variance (ANOVA) and the Duncan’s multiple range method were used to compare significant differences among the groups. The level of significance was set at *p* < 0.05 for all the statistical tests.

## 4. Conclusions

Our results indicated that ginsenoside Rb1 was able to activate Nrf2/ARE pathway in the renal injury induced by IIR, and markedly attenuated IIR induced renal histological and functional injury. Meanwhile, Nrf2 activation was contribute to oxidative stress and tissue damage.
